# Association of Psoriasis Severity with Serum Prolactin, Thyroid Hormones, and Cortisol before and after Treatment

**DOI:** 10.1155/2013/921819

**Published:** 2013-10-28

**Authors:** Reza M. Robati, Parviz Toossi, Mohammad Rahmati-Roodsari, Sara Khalilazar, Ehsan Abolhasani, Nastaran Namazi, Shima Younespour

**Affiliations:** ^1^Skin Research Center, Shahid Beheshti University of Medical Sciences, Shohada-e-Tajrish Hospital, Shahrdari Street, Tajrish Square, Tehran, Iran; ^2^Department of Epidemiology and Biostatistics, School of Public Health, Tehran University of Medical Sciences, Tehran, Iran

## Abstract

*Background*. Prolactin (PRL) level is proposed to be associated with the severity of psoriasis although the previous studies reported different results. *Objective*. To find the association between PRL levels and severity of psoriasis before and after treatment. In addition, we aimed to find a difference in prolactin, thyroid stimulating hormone (TSH), thyroid hormones (T_3_ and T_4_), and cortisol levels between patients with psoriasis and normal controls. *Methods*. First, the levels of hormones were measured in 30 patients with psoriasis and 30 matched controls. The severity was assessed by psoriasis area and severity index (PASI). Then, patients were treated, and PASI was assessed every week until achieving PASI-75 response. At this time, the hormones were measured again and compared to the baseline. *Results*. No statistical significant difference was observed in the mean PRL, T_3_, T_4_, TSH, and cortisol levels between cases and controls. Comparing to the baseline, a significant decrease in PRL levels and a significant increase in T_3_ and serum cortisol levels were observed after treatment (*P* < 0.05), while the changes in other hormones were not significant. *Conclusion*. After treatment, PRL significantly decreased, and T_3_ and cortisol levels significantly increased. No correlation between hormone levels and improvement of PASI score existed.

## 1. Introduction

Psoriasis is a T-cell mediated autoimmune disease [1]. Genetic, environmental, immune defect, and hormonal factors take part in autoimmune pathogenesis of diseases [2]. An environmental factor stimulates cytokines secretion by T-cells that lead to keratinocytes proliferation; in dermal blood vessels, it will also lead to antigenic adhesion molecules production [3]. In addition, some hormones may have role in pathogenesis of psoriasis due to their effects on keratinocytes proliferation; in vitro studies on prolactin (PRL) has demonstrated proliferative effect on keratinocytes, epithelial cells, and lymphocytes. Existence of PRL receptors on epidermal keratinocytes provides more support for the hypothesis stating that PRL might have some role in the etiopathogenesis of psoriasis [4]. Previous studies have assessed the association of PRL levels with the severity of psoriasis; however, the results are contradictory [5–7].

 In addition to PRL, the role of other stress hormones including cortisol, thyroid hormones, and thyroid stimulating hormone (TSH) in psoriasis pathogenesis was investigated previously [8–10]; like PRL, the receptors of thyroid hormones are expressed in the skin [11], and the changes in their levels during the active phase of disease and alleviation of the disease by antithyroid therapy [12, 13] may indicate a correlation between these hormones levels and severity of the disease; cortisol mediates psychoemotional stresses, and it is shown that the cortisol response to stress is diminished in psoriasis [14]. Therefore, it can be assumed that alterations in the activity of hypothalamus-pituitary-adrenal (HPA) axis can have a role in pathogenesis of psoriasis via changes in cortisol levels [10]. 

We conducted a study to answer the following questions: is there any difference in PRL, TSH, T_3_, T_4_, and cortisol levels between psoriatic patients and normal controls? Does the severity of disease correlate with these hormones levels? Do these hormones levels decrease as psoriasis area and severity index (PASI) decrease by treatment? 

## 2. Materials and Methods

We designed a study to assess the association of the severity of psoriasis with changes in the plasma levels of prolactin, TSH, T_3_, T_4_, and cortisol in patients admitted to our university hospital from March 2011 through March 2012. The protocol of the study was designed in accordance with the Helsinki declaration and implemented after receiving approval from the university ethics board. According to our study protocol, we first conducted a study to find the difference in hormone levels between patients and normal controls; then, we followed the patient group until they achieved PASI 75 response by treatment to see whether the decrease in the severity of the disease affected the plasma levels of prolactin. 

 To study the impact of therapy on the patients' serum PRL levels, we decided to conduct a study using paired *t*-test for analysis. Previous studies of this type have had standard deviations in the range of 17 to 34 [5–7]. Therefore, a sample size of 30 would achieve 86% power to detect a difference of −15.0 between the null hypothesis mean (the mean difference between pre- and posttreatment) of 0.0 and the alternative hypothesis mean of 15.0 with an estimated standard deviation of 26.0 and a significance level (alpha) of 0.05 using a two-sided one-sample *t*-test. In addition, 30 age- and sex-matched healthy controls were included in the study. 

 Eligible patients were new cases with clinically and pathologically diagnosed psoriatic lesions who had not received any topical or systemic treatment for their lesions during the last three months. The control participants had no dermatologic or systemic diseases. In both groups, participants with the following conditions were excluded from the study: pregnancy or lactation, menstrual abnormalities, consumption of medications that affected serum PRL levels (i.e., antidepressants, antipsychotics, butyrophenones, estrogens, H2 blockers, methyldopa, metoclopramide, phenothiazines, reserpine, verapamil, etc.), any condition that affected the evaluated hormones levels (including head trauma, prolactinoma, hypothalamus diseases, hypo/hyperthyroidism, and adrenal and renal diseases), and any malignancy or physical and psychiatric condition that could impair participation in the study.

 The patients' treatment protocol was composed of conventional topical and systemic regimens and phototherapy according to the disease severity. To assess the severity of psoriasis, PASI was used before starting treatment and every week until achieving a PASI 75 response. The PASI 75 response is the most commonly used primary efficacy measure, which is the percentage of patients who, at a given point in time, achieve a reduction of at least 75% in their baseline PASI. Other basic characteristic of patients were recorded in a questionnaire. To measure the plasma levels of the hormones in the psoriasis group, five millilitres of blood samples were taken from participants at the baseline and after achieving a PASI 75 response. In order to justify the effect of menstrual changes on prolactin in women, the hormones were measured during the first 7 days of the cycle and before the luteal phase [15]. All samples were collected at 8 a.m. The samples were centrifuged to separate the plasma and stored at −80°C. Then, the samples were transferred to the pathology laboratory of Endocrine Research Centre of the university to measure hormone levels. All the hormonal levels were measured by ELISA (Human ELISA kits, Diametra, Foligno, Italy).

## 3. Statistical Analysis 

The paired *t*-test or Wilcoxon signed-rank test, wherever appropriate, was used to evaluate (1) the differences in the hormonal serum levels before and after achieving PASI 75 and (2) the differences in serum levels of two age- and sex-matched groups. Independent two-sample *t*-test or Mann-Whitney *U* test, wherever appropriate, was employed to assess the differences in serum levels of females and males. Pearson and Spearman correlation tests were used to detect the linear relationships between two variables. All tests applied were two-sided, and the significance level was set at 0.05. All statistical analyses were performed with statistical software SPSS 16.0.0. (SPSS Inc. Chicago, IL, USA). 

## 4. Results 

**Figure 1:**
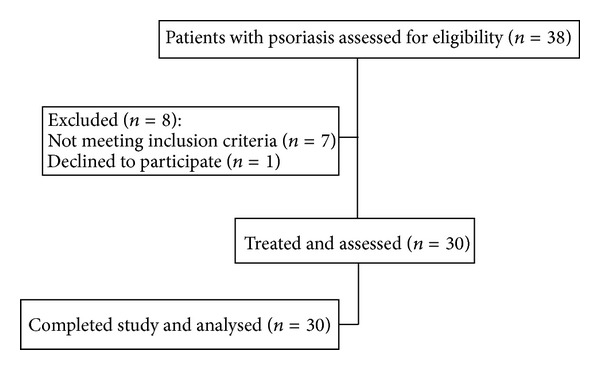
The process of recruiting patients in the psoriasis group.

 We examined 38 new cases of psoriasis; the recruitment process is illustrated in Figure 1. The recruitment process of psoriasis patients is demonstrated in the psoriasis group that comprised 30 patients with psoriasis (14 women and 16 men) and 30 age- and sex-matched healthy controls. The mean ± SD age of patients was 37.5 ± 15.24 (range 21–66 years). The median duration of disease was 9 years with a range of 1 month to 42 years.

 The severity of psoriasis was evaluated before and after the therapy using the PASI score. The mean PASI score was 12.83 ± 10.25 on enrollment (median 11.05, range 2.4–54.0). After the treatment, a significant decrease in disease severity (PASI) was observed (mean PASI 1.97 ± 1.65, median 1.6, range 0–7.7, and *P* < 0.0001). The mean percentage of improvement in PASI scores was 83.48 ± 9.53 (median 82.7, range 65.5–100%). 

 In this study, the mean PRL level was significantly higher in the pretreatment serum of the patients when compared to their posttreatment levels (*P* = 0.04, Table 1). However, no significant difference was observed in the mean level of serum PRL of the cases (before achieving PASI 75) and controls (*P* = 0.64) (Table 1). Also, after achieving PASI 75, the patients' mean serum level of PRL did not differ significantly when compared to healthy controls (*P* = 0.83, Table 1).

 Patients showed a significant increase in their serum cortisol levels after achieving PASI 75 (median cortisol levels were 131 ng/mL at baseline and 146 ng/mL after their therapy, *P* = 0.03). Comparing cases and controls, no significant difference was observed in mean serum cortisol levels of the two groups (*P* = 0.95 and Table 1).

According to Table 1, no difference was observed in the mean levels of thyroid hormones (T_3_, T_4_, and TSH) between cases (before achieving PASI 75) and controls (*P* = 0.80, *P* = 0.13, and *P* = 0.63, resp.). Also, the mean levels of these parameters did not differ significantly between patients (after achieving PASI 75) and controls (*P* values at least 0.31). A significant increase was observed in the patients' plasma levels of T_3_ after the achievement of PASI 75 (the median T_3_ level was 99 ng/dL at baseline and 110 ng/dL after the treatment, *P* = 0.04). However, no significant changes were found in T_4_ and TSH levels after treatment (*P* = 0.31 and *P* = 0.20, resp.). 

 Comparison of the patients and controls revealed no significant differences between females and males in the mean levels of serum PRL, cortisol, thyroid hormones T_3_ and T_4_, and TSH, except for a significantly lower level of prolactin in healthy males (*P*-values at least 0.24) (Table 1). Male patients showed a significant decrease in the mean level of serum PRL after the achievement of PASI 75 (*P* = 0.01) (Table 1). Also, at the beginning of the study, male patients had higher levels of serum PRL in comparison with male controls (*P* = 0.02) (Table 1). There was a significant increase in the male patients' plasma levels of T_3_ after the achievement of PASI 75 (*P* = 0.047). No other significant differences were found in the measured items of male and female participants. 

 No significant associations were observed between the serum PRL and cortisol levels and severity of psoriasis at the beginning of the study. Also, there were no significant correlations between changes in PRL and cortisol levels and the percentage of PASI decrease. Furthermore, no associations were observed between changes in thyroid hormones and PASI improvement. No correlation was found between PRL and cortisol levels of the participants. 

## 5. Discussion

 The association of endocrine system with psoriasis has been a matter of interest for many researchers as it is thought to be an autoimmune disorder that flares up by psychoemotional stress [16, 17]. In our study, the levels of the measured hormones before and after treatment had no difference between patients and controls. Achievement of PASI 75 was accompanied by a significant change in PRL, T_3_, and cortisol levels; however, there was no correlation between changes in the hormonal levels and the PASI score changes. In addition, there was no difference between genders with regard to changes in the hormonal levels. On the other hand, comparison of the male patients and male controls showed that their PRL level was significantly higher before treatment and after achieving PASI 75; however, they had no significant difference in the level of this hormone. PRL levels are higher in male patients in comparison with control group and returns to normal by treatment. Yet, our study could not determine whether PRL had a causative role in psoriasis in males. 

 In our study, no significant differences were observed in PRL levels between psoriasis patients and normal controls, which was similar to the results of the study performed by Gorpelioglu et al. [7]; however, the PRL levels have been reported to be higher in psoriasis patients in some other studies [5, 18]. In our study, no association was found between PRL and the severity of psoriasis. The association of PRL levels with the severity of psoriasis is another issue of controversy; some reports have claimed no correlation [7] while some other studies have reported a significant positive correlation [18]. The contradictory results in different studies might be due to the local production of PRL; in a study by El-Khateeb et al. [19], a significant difference was reported between PRL levels of the psoriasis lesional skin and serum or nonlesional skin. This finding can justify the lack of difference in PRL levels in our study; in addition, the difference found in some studies might be due to enrolling more severe cases. 

 In our study, a significant increase was seen in total T_3_ after achieving PASI 75 response while T_4_ and TSH did not change significantly. Different reports with regard to these hormones are available; some show higher levels of both T_4_ and free T_3_ while the results of some other studies indicate higher levels of T_4_ with no association with severity [8–10]. The receptors of T_3_ are expressed in epidermal keratinocytes [11] and may have a role in the proliferation of these cells by increasing epidermal growth factor (EGF); the EGF receptors expression is increased in psoriasis [20] that may highlight the effect of thyroid hormones in the pathogenesis of psoriasis. Previous reports of successful treatment of psoriasis by antithyroid medications is another evidence of their role in psoriasis pathogenesis; however, the mechanism of action is unknown [12, 13]. 

The levels of cortisol increased in our patients after achieving PASI 75 response, which shows the important role of low cortisol levels in the pathogenesis of psoriasis. In other words, low cortisol levels predispose patients with psoriasis to exacerbations by psychoemotional stress. Alterations in the activity of hypothalamus-pituitary-adrenal (HPA) axis might play a role in the pathogenesis of psoriasis [10, 14]. In addition, the effect of cortisol on the balance of T-helper type 1 (TH1) and type 2 (TH2) lymphocytes provides evidence for the role of this hormone in the pathogenesis of this disease by dysregulation of the TH1/TH2 balance [21, 22]. The immunomodulatory effects of cortisol in pathogenesis of psoriasis are highlighted by the fact that the earliest immunologic event in new psoriasis lesions is accumulation of CD4+ and CD8+ T-helper lymphocyte and extravasation of T-helper lymphocytes [23].

One limitation to our study was that we could not measure the level of stress in each patient; however, this variable was randomly distributed among our participants and none of them was under obvious stress at the time of hormonal measurement. In addition, patients with psoriasis frequently experience exacerbation and remission and are on treatment regimens most of the time; hence, finding patients who were not on therapeutic regimens and new cases of psoriasis was a big challenge and the reason for a smaller sample size. We focused on serum levels of PRL and other hormones; however, future studies might measure these hormonal levels in the skin and evaluate the expression of their receptors simultaneously. In conclusion, we found no correlation between hormones levels and the severity of psoriasis. Furthermore, according to our results, the only hormone that increases in psoriasis is PRL in male patients, which returns to the same level as healthy controls after treatment.

## Figures and Tables

**Table 1:** Summary of serum levels of PRL, cortisol, and thyroid hormones based on study groups.

	Cases (*n* = 30)		Controls (*n* = 30)
	Before treatment (*n* = 30)	After treatment (*n* = 27)	
Prolactin^a^, (ng/mL)	14.08 ± 7.36; 11.80 (4.8–41.2)	11.57 ± 5.00; 11.00 (5–23.2)	13.14 ± 10.09; 10.65 (3.9–41.9)
Female	14.22 ± 9.06; 11.45 (4.8–41.2)	11.60 ± 3.29; 11.90 (6.4–17.3)	16.74 ± 13.37; 10.85 (4.0–41.9)
Male	13.96 ± 5.78; 12.90 (5.3–22.6)	11.54 ± 6.16; 8.40 (5–23.2)	9.99 ± 4.36; 10.65 (3.9–21.2)
Cortisol^b^, (ng/mL)	146.07 ± 63.66; 131 (67–387)	169.04 ± 61.14; 146 (91–292)	147.72 ± 67.64; 134 (59–313)
Female	133.43 ± 41.85; 127 (67–228)	158.75 ± 54.35; 139.5 (100–263)	133.31 ± 59.86; 131 (67–313)
Male	157.12 ± 77.71; 133.5 (80–387)	177.27 ± 66.77; 146 (91–292)	159.44 ± 73.12; 141.5 (59–300)
T_3_^c^, (ng/dL)	105.30 ± 19.99; 99 (77–159)	111.11 ± 16.74; 110 (89–159)	106.90 ± 23.84; 99 (72–159)
Female	105.93 ± 23.42; 97 (84–159)	108.17 ± 14.76; 110.5 (89–133)	106.07 ± 31.36; 95 (72–159)
Male	104.75 ± 17.22; 99 (77–138)	113.47 ± 18.33; 110 (91–159)	107.62 ± 15.67; 99 (86–136)
T_4_, (mg/dL)	9.48 ± 1.65; 9.50 (6–13.1)	9.18 ± 1.49; 9.20 (5.9–12.3)	8.93 ± 1.19; 8.60 (6.5–10.9)
Female	9.52 ± 1.78; 9.50 (7.4–13.1)	9.55 ± 1.26; 9.20 (8–12.3)	8.81 ± 0.95; 8.60 (7.5–10.9)
Male	9.44 ± 1.58; 9.65 (6–12)	8.91 ± 1.62; 9.20 (5.9–11.2)	9.03 ± 1.39; 8.95 (6.5–10.7)
TSH, (mIu/L)	2.82 ± 1.96; 2.28 (0.5–8.2)	2.07 ± 1.25; 1.76 (0.9–6.6)	2.65 ± 1.63; 2.67 (0.8–6.8)
Female	2.80 ± 1.92; 2.58 (0.5–7.3)	2.23 ± 1.13; 2.02 (0.9–4.6)	2.65 ± 1.30; 2.79 (0.8–5.0)
Male	2.84 ± 2.07; 1.99 (0.9–8.2)	1.94 ± 1.36; 1.66 (1.1–6.6)	2.64 ± 1.91; 2.28 (0.8–6.8)

Data are expressed as mean ± SD; median (range).

^a^The mean PRL level was significantly higher in the pretreatment serum of the patients compared with their posttreatment levels (paired *t*-test, *P* = 0.04).

^b^Patients had a significant increase in their serum cortisol levels after achieving PASI 75 (Wilcoxon signed-rank test, *P* = 0.03).

^c^A significant increase was observed in plasma T_3_ levels of patients after achievement of PASI 75 (Wilcoxon signed-rank test, *P* = 0.04).
